# Steering an Age-Friendly University (AFU) Initiative: Insights from Directors of Aging Centers

**DOI:** 10.1093/geroni/igaa057.1789

**Published:** 2020-12-16

**Authors:** Joann Montepare, Kimberly Farah

## Abstract

The pioneering Age-Friendly University (AFU) initiative, endorsed in 2016 by GSA’s Academy for Gerontology in Higher Education (AGHE), calls for institutions of higher education to respond to shifting demographics and the needs of our aging populations through more age-friendly programs, practices, and partnerships. Over 65 institutions have joined the AFU network and adopted the 10 AFU guiding principles. This symposium will feature leaders at AFU campuses representing centers on aging and gerontology programs who will discuss why their institution joined the initiative, their age-friendly campus vision, and their recommendations for mounting an AFU initiative. AFU Director Charness (Florida State University) will describe launching an AFU initiative from the perspective of a state university, with a focus on critical discussion points, the concerns of upper administration, and the timeline for completion of the process. AFU Director Porter (University of Manitoba) will discuss how the Centre on Aging has been using its mandate as a research centre to advance the age-friendly movement through research, knowledge mobilization, training, and partnership initiatives. AFU Director Schumacher (University of Maryland Baltimore County) will talk about how the AFU framework has served as a platform for new and constructive synergies among gerontology programs, centers on aging, health systems, and higher education. AFU Director Gugliucci (University of New England) will discuss strategies for leading health professions education programs within an AFU framework, with special attention to AGHE resources available to support these efforts. Directors of Aging Centers Interest Group Sponsored Symposium.

